# Redescription of the little-known grasshopper *Willemsella* (Acrididae, Hemiacridinae) from Peninsular Malaysia

**DOI:** 10.3897/BDJ.4.e7775

**Published:** 2016-03-07

**Authors:** Ming Kai Tan, Khairul N Kamaruddin

**Affiliations:** ‡ National University of Singapore, Singapore, Singapore; § Ex-situ Conservation Division, Department of Wildlife and National Parks, Kuala Lumpur, Malaysia

**Keywords:** Orthoptera, Catantopinae, male phallic complex, new locality record, Southeast Asia

## Abstract

**Background:**

Catantopinae is a huge subfamily and is in need of a major revision. We contribute to the taxonomy of the subfamily by reviewing one poorly known genus, *Willemsella* Miller, 1934. This is a monotypic genus so far found only in Peninsular Malaysia.

**New information:**

Rediscovery and new locality records of the species was reported here. The species is redescribed in accordance to modern criteria of taxonomy. Male phallic complex is also described for the first time. The subfamily position of the species is also verified and is more correctly placed under Hemiacridinae.

## Introduction

Catantopinae is a huge subfamily consisting of around 16 tribes and about 382 genera and is distributed throughout the old world (Dirsh 1961, Eades et al. 2015). Catantopinae is in need of a major revision because it has traditionally been a “dumping group” for species requiring further study (Dirsh 1961). It is also for this reason that Catantopinae is an extremely difficult group to work on. Many genera within Catantopinae still require clarification of their taxonomic positions and cumulative works over many years may be required. Here, we hope to contribute to the taxonomy of the subfamily by reviewing one of the lesser known genus, *Willemsella* Miller, 1934.

*Willemsella* is a monotypic genus so far and is found only in Peninsular Malaysia. Described in 1934 based on a pair of copulating adults in Dusan Tua, Selangor (Miller 1934), there was no record of *Willemsella bicolor* Miller, 1934 since then (Eades et al. 2015). It was only in recent years (after 2012) that this species was rediscovered in a new locality, Bukit Fraser, Pahang (Tan and Kamaruddin 2014). Since then, more specimens were collected from Bukit Larut, Perak, suggesting that this species is more widespread than previously known.

While the original description of the genus and its type species is substantial, description of the male phallic complex is lacking. It was also not examined in the major revision of phallic complexes of grasshoppers (Dirsh 1956, Dirsh 1961, Dirsh 1975. Miller's description (Miller 1934) came without a diagnosis. Here, we also provide the first diagnosis for the species. New close-up photographs using high-definition imaging system are also necessary for clearer illustration of the species. This is essential to verify and establish the subfamilial position of this poorly known genus. We redescribe the species including description and illustration of the male phallic complex. We also provided an updated distribution data for the monotypic genus.

## Materials and methods

New material was collected by the authors in the highlands of Peninsular Malaysia: Bukit Fraser (Pahang) from 2012 to 2013 and Bukit Larut (Perak) in 2015. The grasshoppers were mainly collected by opportunistic collection and sweep netting among tall grasses in the day or night. The specimens were either preserved in absolute analytic alcohol and/ or air dried and pinned. Photographic images were done with a Canon EOS 500D digital SLR camera with a compact-macro lens EF 100mm 1:2.8 USM, then edited using Adobe Photoshop. Images of the male genitalia were done using the Visionary Digital System. Scales are given with the images. Male genitalia were preserved in glycerine. Whenever possible, in-situ images were also taken using a Canon EOS 500D digital SLR camera with a compact-macro lens. Measurements of dried pinned specimen were made using Vernier calipers. In the measurement section, the following terms were used: BL = body length; VW = vertex width between eyes; FRW = frontal ridge width between antennae; PL = pronotum length; PZL = prozona length; MZL = metazona length; PW = pronotum width; TL = tegmen length; HFL = hind femur length; HFW = hind femur width (maximum); HTL = hind tibia length. The specimens are deposited in the Zoological Reference Collection (ZRC), Lee Kong Chian Natural History Museum (former Raffles Museum of Biodiversity Research).

For description and illustration captures, we use the following terms as already used by Dirsh 1975:

Epiphallus:

B Bridge

A Ancora

An Anterior projection

L Lophus

Ectophallus:

Dsv Dorsal ectophallic sclerite

Esv Ventral ectophallic sclerite

Apd Apodeme

Ac Arch of cingulum

Ap Apical valve of penis (= aedeagus)

Bp Basal valve of penis (= endoparameres, endophallic plates)

## Taxon treatments

### Willemsella bicolor

Miller, 1934

Willemsella bicolor The genus should be placed more correctly under Hemiacridinae, rather than Catantopinae. In *Willemsella*, the basal and apical penis valves are separated, diagnostic for species in Hemiacridinae. In Catantopinae, the basal and penis valves are connected. The genus also exhibits a series of regular transverse veinlets near the radial area of tegmen, similar to those found in Hemiacridinae and usually absent in Catantopinae. *Willemsella* also shares characters of Catantopinae: presence of prosternal spine, rounded mesosternal lobes and absence of intercalary veins in the medial area of tegmen.

#### Materials

- **Type status:** Other material. **Occurrence:** catalogNumber: LAR.15.53; recordedBy: Ming Kai Tan et al.; individualCount: 1; sex: male; lifeStage: adult; disposition: ZRC; **Taxon:** scientificName: Willemsella bicolor; nameAccordingTo: Miller; namePublishedIn: 1934; kingdom: Animalia; phylum: Arthropod; class: Insecta; order: Orthoptera; family: Acrididae; genus: Willemsella; specificEpithet: bicolor; **Location:** country: Peninsular Malaysia; stateProvince: Perak; locality: Bukit Larut (Maxwell Hill); verbatimElevation: 1056 m; decimalLatitude: 4.86184; decimalLongitude: 100.79276; **Identification:** identifiedBy: M. K. Tan; dateIdentified: 2015; **Event:** samplingProtocol: active collecting; eventDate: 06/18/2015; eventTime: 1053-1201
- **Type status:** Other material. **Occurrence:** catalogNumber: LAR.15.189; recordedBy: Ming Kai Tan et al.; individualCount: 1; sex: male; lifeStage: adult; disposition: ZRC; **Taxon:** scientificName: Willemsella bicolor; nameAccordingTo: Miller; namePublishedIn: 1934; kingdom: Animalia; phylum: Arthropod; class: Insecta; order: Orthoptera; family: Acrididae; genus: Willemsella; specificEpithet: bicolor; **Location:** country: Peninsular Malaysia; stateProvince: Perak; locality: Bukit Larut (Maxwell Hill); verbatimElevation: 920.4 m; decimalLatitude: 4.86626; decimalLongitude: 100.78865; **Identification:** identifiedBy: M. K. Tan; dateIdentified: 2015; **Event:** samplingProtocol: active collecting; eventDate: 09/19/2015; eventTime: 1925-2049
- **Type status:** Other material. **Occurrence:** catalogNumber: LAR.15.199; recordedBy: Ming Kai Tan et al.; individualCount: 1; sex: male; lifeStage: adult; disposition: ZRC; **Taxon:** scientificName: Willemsella bicolor; nameAccordingTo: Miller; namePublishedIn: 1934; kingdom: Animalia; phylum: Arthropod; class: Insecta; order: Orthoptera; family: Acrididae; genus: Willemsella; specificEpithet: bicolor; **Location:** country: Peninsular Malaysia; stateProvince: Perak; locality: Bukit Larut (Maxwell Hill); verbatimElevation: 1001 m; decimalLatitude: 4.86289; decimalLongitude: 100.79163; **Identification:** identifiedBy: M. K. Tan; dateIdentified: 2015; **Event:** samplingProtocol: active collecting; eventDate: 09/20/2015; eventTime: 1043-1236
- **Type status:** Other material. **Occurrence:** catalogNumber: LAR.15.203; recordedBy: Ming Kai Tan et al.; individualCount: 1; sex: male; lifeStage: adult; disposition: ZRC; **Taxon:** scientificName: Willemsella bicolor; nameAccordingTo: Miller; namePublishedIn: 1934; kingdom: Animalia; phylum: Arthropod; class: Insecta; order: Orthoptera; family: Acrididae; genus: Willemsella; specificEpithet: bicolor; **Location:** country: Peninsular Malaysia; stateProvince: Perak; locality: Bukit Larut (Maxwell Hill); verbatimElevation: 1097 m; decimalLatitude: 4.86167; decimalLongitude: 100.79434; **Identification:** identifiedBy: M. K. Tan; dateIdentified: 2015; **Event:** samplingProtocol: active collecting; eventDate: 09/20/2015; eventTime: 1928-2055
- **Type status:** Other material. **Occurrence:** catalogNumber: LAR.15.210; recordedBy: Ming Kai Tan et al.; individualCount: 1; sex: female; lifeStage: adult; disposition: ZRC; **Taxon:** scientificName: Willemsella bicolor; nameAccordingTo: Miller; namePublishedIn: 1934; kingdom: Animalia; phylum: Arthropod; class: Insecta; order: Orthoptera; family: Acrididae; genus: Willemsella; specificEpithet: bicolor; **Location:** country: Peninsular Malaysia; stateProvince: Perak; locality: Bukit Larut (Maxwell Hill); verbatimElevation: 1097 m; decimalLatitude: 4.86167; decimalLongitude: 100.79434; **Identification:** identifiedBy: M. K. Tan; dateIdentified: 2015; **Event:** samplingProtocol: active collecting; eventDate: 09/20/2015; eventTime: 1928-2055
- **Type status:** Other material. **Occurrence:** catalogNumber: LAR.15.211; recordedBy: Ming Kai Tan et al.; individualCount: 1; sex: female; lifeStage: adult; disposition: ZRC; **Taxon:** scientificName: Willemsella bicolor; nameAccordingTo: Miller; namePublishedIn: 1934; kingdom: Animalia; phylum: Arthropod; class: Insecta; order: Orthoptera; family: Acrididae; genus: Willemsella; specificEpithet: bicolor; **Location:** country: Peninsular Malaysia; stateProvince: Perak; locality: Bukit Larut (Maxwell Hill); verbatimElevation: 1052 m; decimalLatitude: 4.86191; decimalLongitude: 100.79305; **Identification:** identifiedBy: M. K. Tan; dateIdentified: 2015; **Event:** samplingProtocol: active collecting; eventDate: 09/20/2015; eventTime: 2055-2136
- **Type status:** Other material. **Occurrence:** catalogNumber: FRA.12.88; recordedBy: Ming Kai Tan et al.; individualCount: 1; sex: male; lifeStage: adult; disposition: ZRC; **Taxon:** scientificName: Willemsella bicolor; nameAccordingTo: Miller; namePublishedIn: 1934; kingdom: Animalia; phylum: Arthropod; class: Insecta; order: Orthoptera; family: Acrididae; genus: Willemsella; specificEpithet: bicolor; **Location:** country: Peninsular Malaysia; stateProvince: Pahang; locality: Bukit Fraser; verbatimElevation: 1214 m; decimalLatitude: 3.71944; decimalLongitude: 101.72936; **Identification:** identifiedBy: M. K. Tan; dateIdentified: 2015; **Event:** samplingProtocol: active collecting; eventDate: 12/27/2012; eventTime: 1338
- **Type status:** Other material. **Occurrence:** catalogNumber: FRA.12.90; recordedBy: Ming Kai Tan et al.; individualCount: 1; sex: male; lifeStage: adult; disposition: ZRC; **Taxon:** scientificName: Willemsella bicolor; nameAccordingTo: Miller; namePublishedIn: 1934; kingdom: Animalia; phylum: Arthropod; class: Insecta; order: Orthoptera; family: Acrididae; genus: Willemsella; specificEpithet: bicolor; **Location:** country: Peninsular Malaysia; stateProvince: Pahang; locality: Bukit Fraser; verbatimElevation: 1203 m; decimalLatitude: 3.71952; decimalLongitude: 101.72937; **Identification:** identifiedBy: M. K. Tan; dateIdentified: 2015; **Event:** samplingProtocol: active collecting; eventDate: 12/27/2012; eventTime: 1346
- **Type status:** Other material. **Occurrence:** catalogNumber: FRA.12.112; recordedBy: Ming Kai Tan et al.; individualCount: 1; sex: male; lifeStage: adult; disposition: ZRC; **Taxon:** scientificName: Willemsella bicolor; nameAccordingTo: Miller; namePublishedIn: 1934; kingdom: Animalia; phylum: Arthropod; class: Insecta; order: Orthoptera; family: Acrididae; genus: Willemsella; specificEpithet: bicolor; **Location:** country: Peninsular Malaysia; stateProvince: Pahang; locality: Bukit Fraser; verbatimElevation: 1209 m; decimalLatitude: 3.71943; decimalLongitude: 101.72935; **Identification:** identifiedBy: M. K. Tan; dateIdentified: 2015; **Event:** samplingProtocol: active collecting; eventDate: 12/29/2012; eventTime: 2005
- **Type status:** Other material. **Occurrence:** catalogNumber: FRA.13.35; recordedBy: Ming Kai Tan et al.; individualCount: 1; sex: female; lifeStage: adult; disposition: ZRC; **Taxon:** scientificName: Willemsella bicolor; nameAccordingTo: Miller; namePublishedIn: 1934; kingdom: Animalia; phylum: Arthropod; class: Insecta; order: Orthoptera; family: Acrididae; genus: Willemsella; specificEpithet: bicolor; **Location:** country: Peninsular Malaysia; stateProvince: Pahang; locality: Bukit Fraser; verbatimElevation: 1227 m; decimalLatitude: 3.71836; decimalLongitude: 101.72987; **Identification:** identifiedBy: M. K. Tan; dateIdentified: 2015; **Event:** samplingProtocol: active collecting; eventDate: 05/19/2013; eventTime: 1409

#### Description

Adult habitus as shown in Fig. 1. Head about as long as pronotum (Fig. 2a, b, d, e). Frontal ridge narrow, parallel-sided dorsal and ventral of median ocellus; ridge more deep but narrower in males than females (Fig. 2c, f). Fastigium of vertex broadly rounded to subtruncated (Fig. 2b, e). Vertex between eyes average about 4.3 times in males (n=3, min=3.5, max=5.0) and 2.0 times (n=2, min=2.0, max=2.0) in females as broad as frontal ridge between antennae. Eyes large. Antennae about 20-segmented, flattened; reaching and surpassing posterior margin of pronotum in males, and reaching middle but not surpassing posterior margin of pronotum in females. Pronotum long, crossed by three transverse furrows (Fig. 2b, e); prozona 1.7 times in male (n=3, min=1.5, max=2.1) and 2.1 times in female (n=2, min=1.9, max=2.4) as long as metazona; anterior margin of prozona truncated to feebly convex; posterior margin of metazona broadly rounded; lateral carinae absent; median carina indistinct, more distinct along metazona. Prosternal spine distinct, flattened and triangular basally, narrowing into a long and subacute apex. Mesosternal lophi with internal margin broadly rounded; mesosternal interspace slightly less broad than lobe. Metasternal lophi with internal margin broadly rounded, separated feebly by metasternal interspace. Tegmen truncated with rounded apex, overlapping each other in rest position, elongated and reaching base of epiproct in males and base of 8^th^ tergite in females. Radial area of tegmen with a series of regular transverse veinlets (Fig. 2a, d). Hind wings concealed under tegmen. Hind femora 4.7 times (n=5, min=4.6, max=4.8) as long as maximal width of these femora; dorsal carina smooth; knee with dorsal external lobe subtruncated to angular. Hind femora 1.1 times (n=5, min=1.1, max=1.1) longer than hind tibiae. Hind tibiae setose, with 8 outer dorsal subapical spines and 8 inner dorsal subapical spines; with 2 outer and 2 inner apical dorsal spines. Hind tarsi about a third of hind tibiae; third segment (without claws) longer than the two basal segments together; arolium large, almost reaching apex of claws.

Male. Epiproct broadly tongue-shaped with an apical lobe; median sulcus shallow, broad and feeble, only in the basal half bordered by broad parallel carinae; apical lobe triangular with obtuse apex (Fig. 3a). Cercus in dorsal view compressed and gently curved externally; apex obtusely rounded. Subgenital plate conical, with obtuse apex (Fig. 3a). Male phallic complex as in Fig. 3b, c. Epiphallus bridge (B), very broad and stout, slightly curved along apical margin. Anchora (A) articulated, extending in less than 90º-angle to epiphallus bridge, apex obtuse from dorsal view; membrane between lophi sparsely granulated. Anterior projection (An) of epiphallus swollen and somewhat sparsely granular, forming at less than 90º-angle to the anchora. Lophi (L) stout and transverse, forming highly sclerotized lateral angles; internal angle bulbous and granular, external angle slightly flattened and obtuse, less sclerotized and granular. Ectophallic membrane thickened to form a dorsal and paired ventral ectophallic sclerites; dorsal ectophallic sclerite (Dsv) more membraneous, bridge-shaped with paired cranial appendages covering the cingulum dorsally and laterally; ventral appendages ventral ectophallic sclerites (Esv) rounded, sclerotized and granular. Apodemes (Apd) robust, weakly sclerotized, extending to basal end. Arch of cingulum (Ac) sclerotized, more so internally; finely granulated externally. Basal and apical penis valves separated (Fig. 3c​). Basal valves of penis (Bp) broadening basally, more sclerotized internally; apical valves (Ap) truncated and sclerotized, slightly surpassing arch of cingulum.

Female. Tenth abdominal tergite with posterior margin rounded-triangularly excised, without lateral lobe (Fig. 3d). Supra–anal plate elongated, separated into two parts by a middle transverse carina, apical half broadly tongue-shaped with obtuse apex (Fig. 3d). Transverse carina of supra-anal plate continuing laterally. Cercus triangular with pointed apex. Subgenital plate rectangular slightly longer than broad; posterior margin with large median angular projection and without lateral excisions (Fig. 3f). Ovipositor with long setae, short, hook-like; dorsal valves smooth with apex gently curved dorsad; ventral valves with apex bent ventrad (Fig. 3e).

Colouration (Fig. 1). Male generally brightly green, female brown to olive brown. Head in male mostly green with some patches of yellow beneath eyes at gena and frons; in females, brown dorsally and anteriorly with some dark olive green patch, dark olive green laterally. Scapus and pedicel black, sometimes with white ring apically. Antennal segments bright red. Disc of pronotum green in male and brown in female; lateral lobes with dorsal third dark green in male or dark olive brown in female, basally with a bright yellow spot extending from the anterior to the posterior margins of lateral lobe. Tegmen green in male and brown in female. Fore and mid femora and tibiae green, with knees black, tarsi dark in male; brown in female. Hind femur generally green in male and brown in female; dorso-basal area yellow, ventro-internal bright red; hind knee black. Hind tibia dark blue. Abdominal tergite green in male or brown in female with dark and pale yellow spots. Thoracic and abdominal sternites bright red.

Measurement of alcohol-preserved dry-pinned specimens, in mm (mean in bracket). Male (n=3) BL: 18.4-19.9 (19.1), VW: 0.5-0.7 (0.6), FRW: 0.1-0.2 (0.1), PL: 3.4-4.2 (3.8), PZL: 2.3-2.6 (2.4), MZL: 1.1-1.6 (1.4), PW: 3.0-3.4 (3.2), TL: 7.9-8.7 (8.4), HFL: 12.7-13.6 (13.1), HFW: 2.7-2.9 (2.8), HTL: 11.5-12.5 (12.0); female (n=2) BL: 21.9-22.4 (22.2), VW: 1.0, FRW: 0.5, PL: 5.2-5.5 (5.4), PZL: 3.4-3.9 (3.7), MZL: 1.6-1.8 (1.7), PW: 4.1-4.6 (4.4), TL: 9.9-10.2 (10.1), HFL: 15.4-15.9 (15.7), HFW: 3.2-3.4 (3.3), HTL: 14.0-14.2 (14.1).

#### Diagnosis

Prosternal spine distinct, flattened and triangular basally, narrowing into a long and subacute apex. Radial area of tegmen with a series of regular transverse veinlets. Anchora (A) articulated, extending in less than 90º-angle to epiphallus bridge, apex obtuse from dorsal view; membrane between lophi sparsely granulated. Anterior projection (An) of epiphallus swollen and somewhat sparsely granular, forming at less than 90º-angle to the anchora. Basal and apical penis valves separated. Antennal segments bright red. Lateral lobes with a basal bright yellow spot extending from the anterior to the posterior margins of lateral lobe.

#### Distribution

Malay Peninsula: Bukit Larut, Perak; Bukit Fraser, Pahang; Dusan Tua, Selangor (Miller 1934).

#### Ecology

Little is known about the ecology of this grasshopper species. they are fairly common among tall grasses in Bukit Fraser and Bukit Larut, suggesting that they may prefer high elevations.

#### Conservation

So far endemic to Peninsular Malaysia.

## Supplementary Material

XML Treatment for Willemsella bicolor

## Figures and Tables

**Figure 1a.**
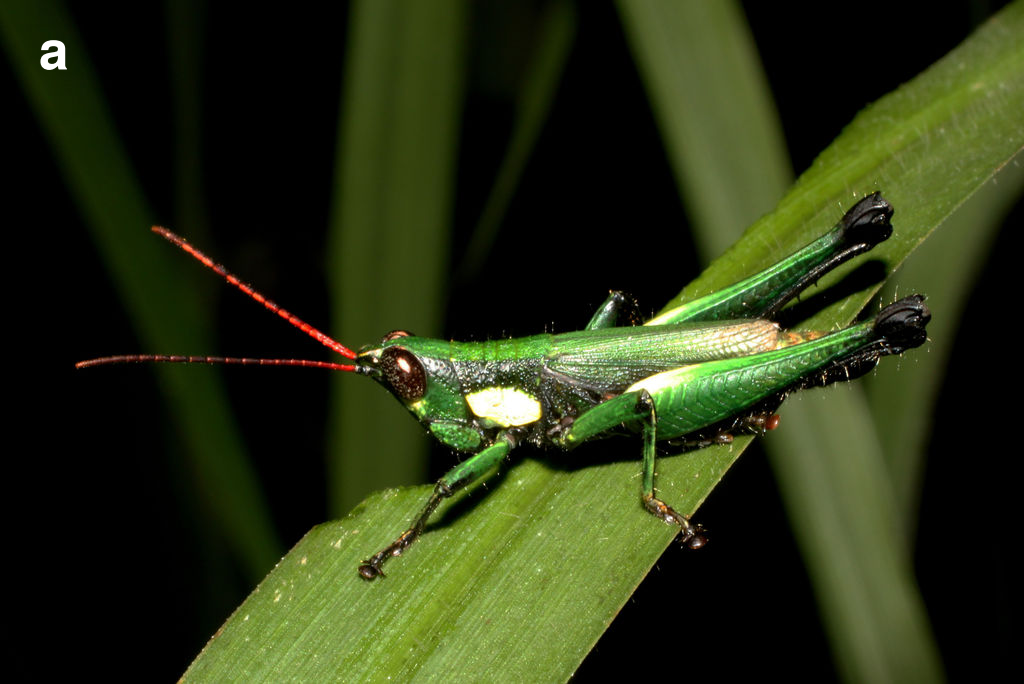
Male adult.

**Figure 1b.**
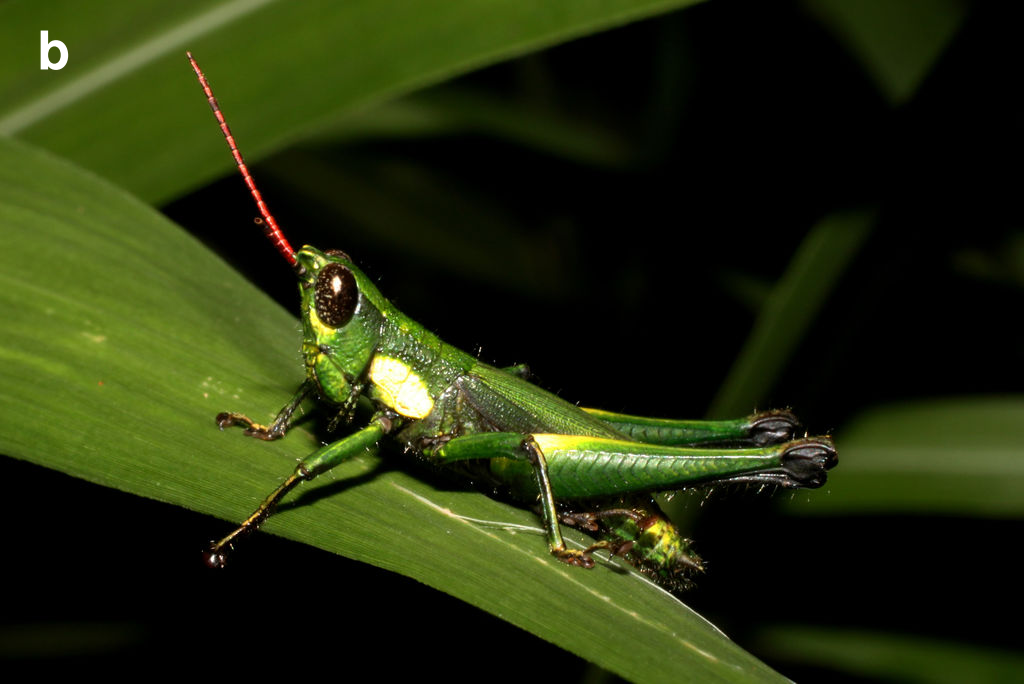
Male adult.

**Figure 1c.**
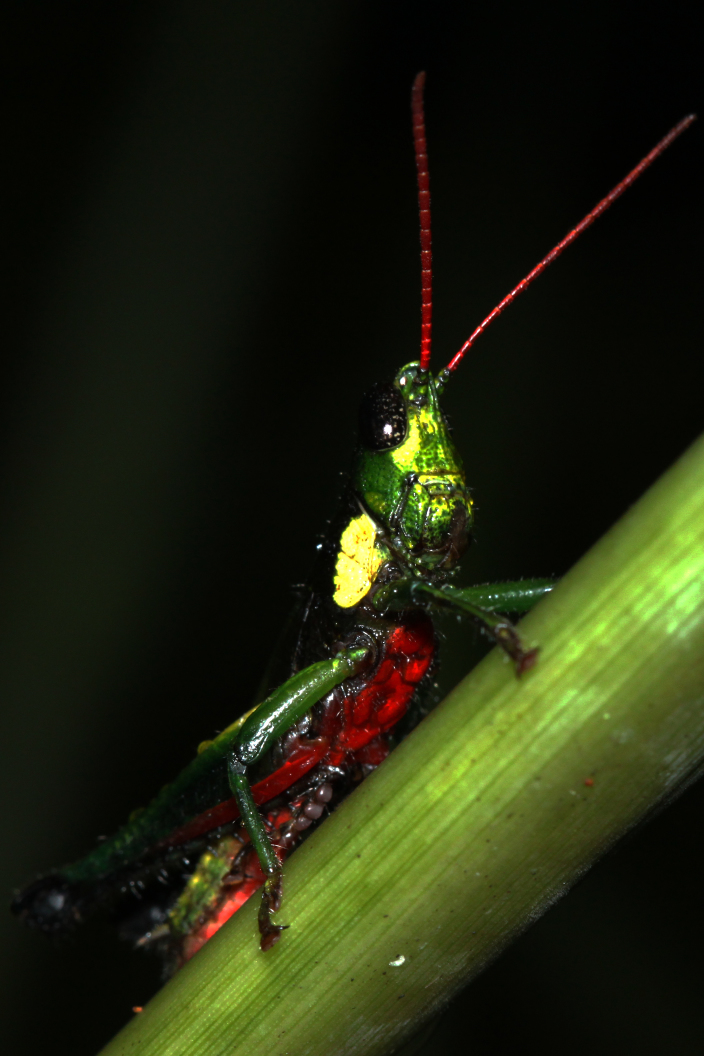
Male adult.

**Figure 1d.**
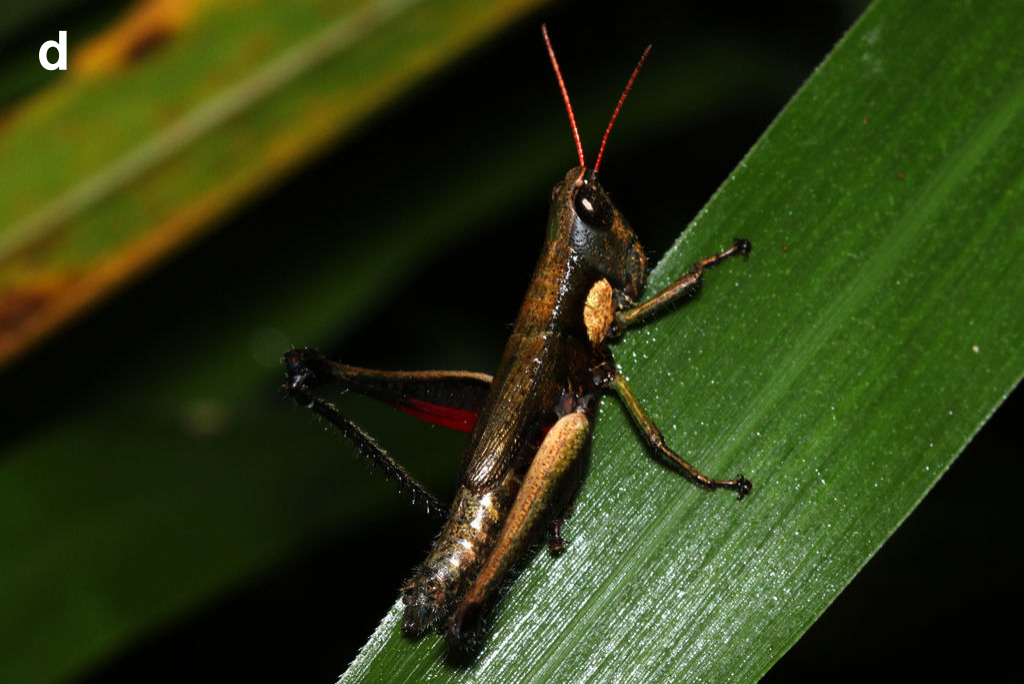
Female adult.

**Figure 2a.**
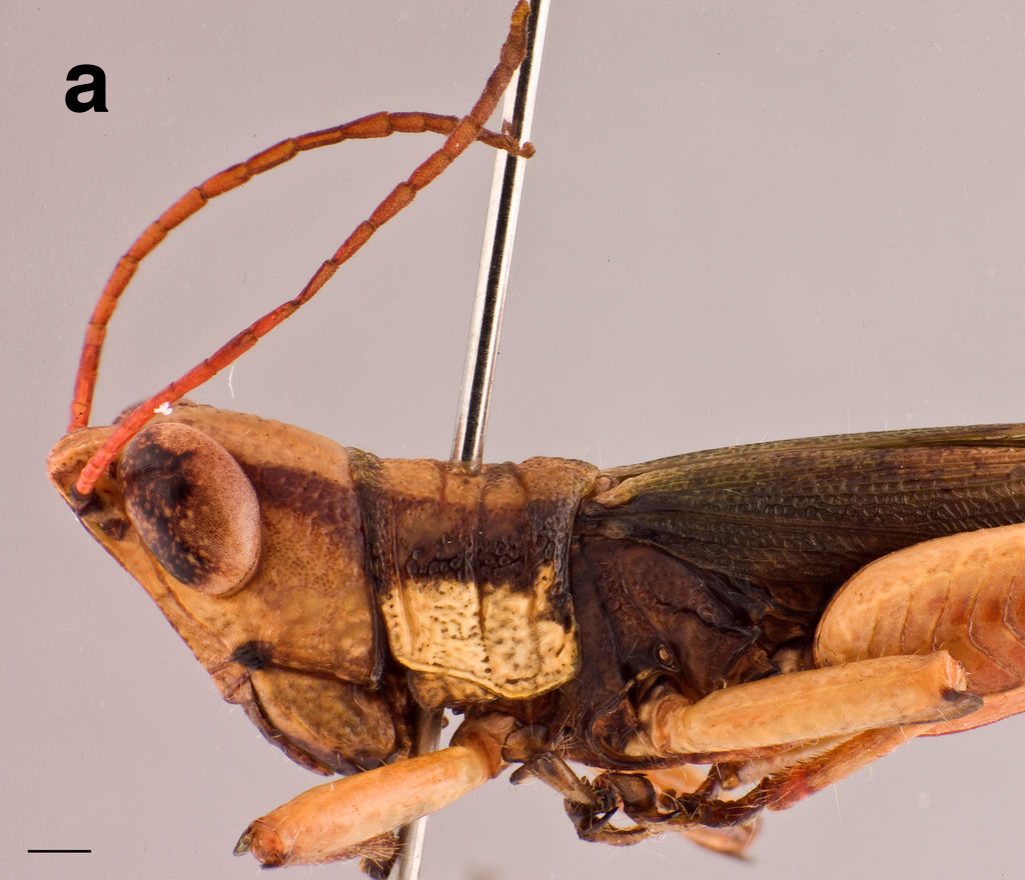
Male head and pronotum in lateral view.

**Figure 2b.**
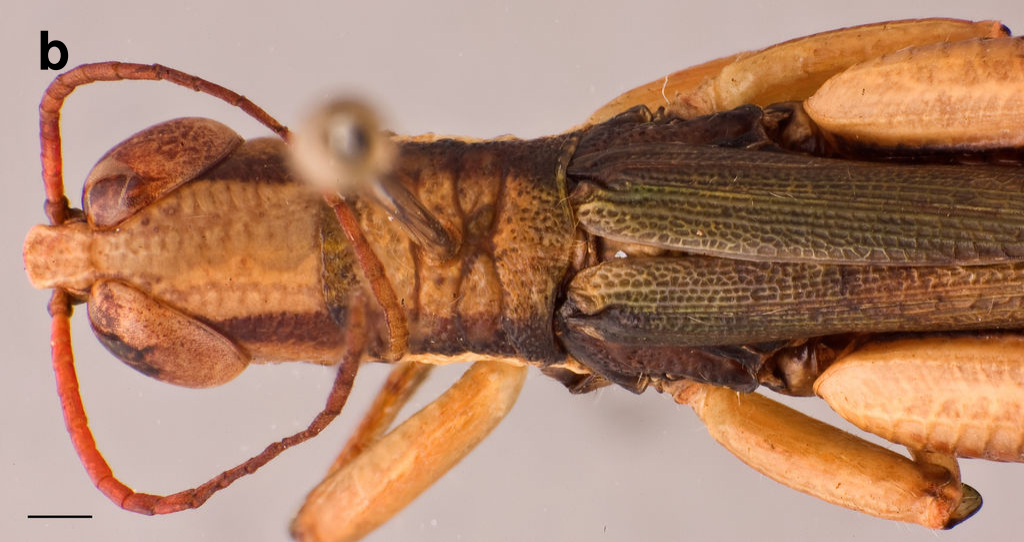
Male head and pronotum in dorsal view.

**Figure 2c.**
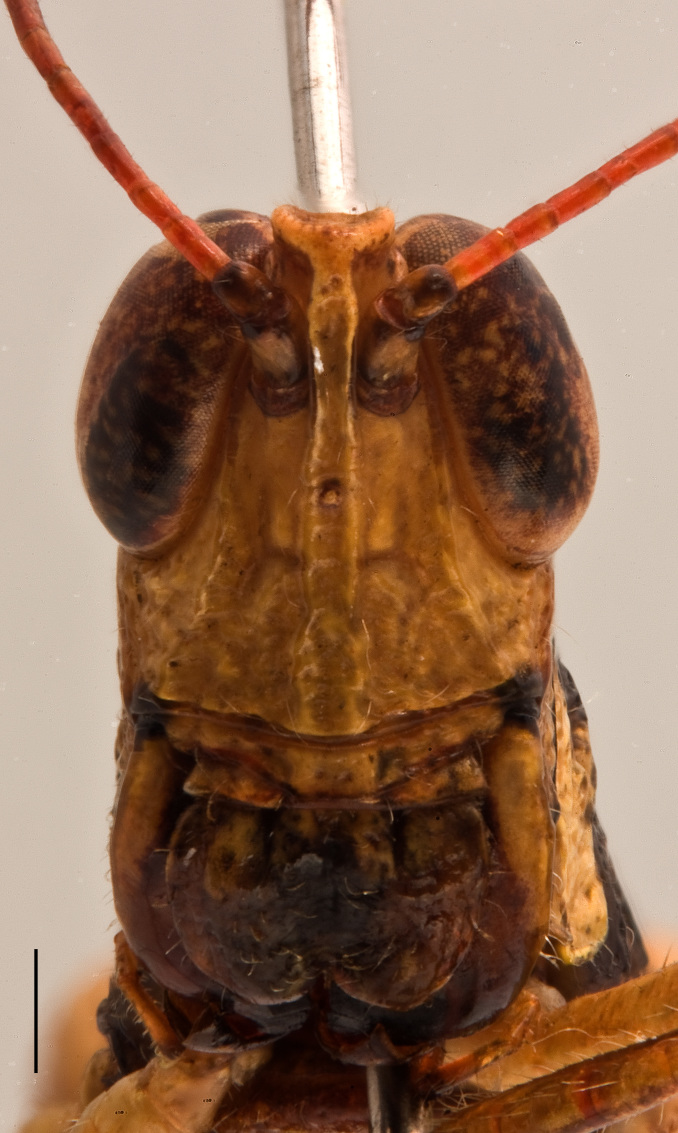
Male face in anterior view.

**Figure 2d.**
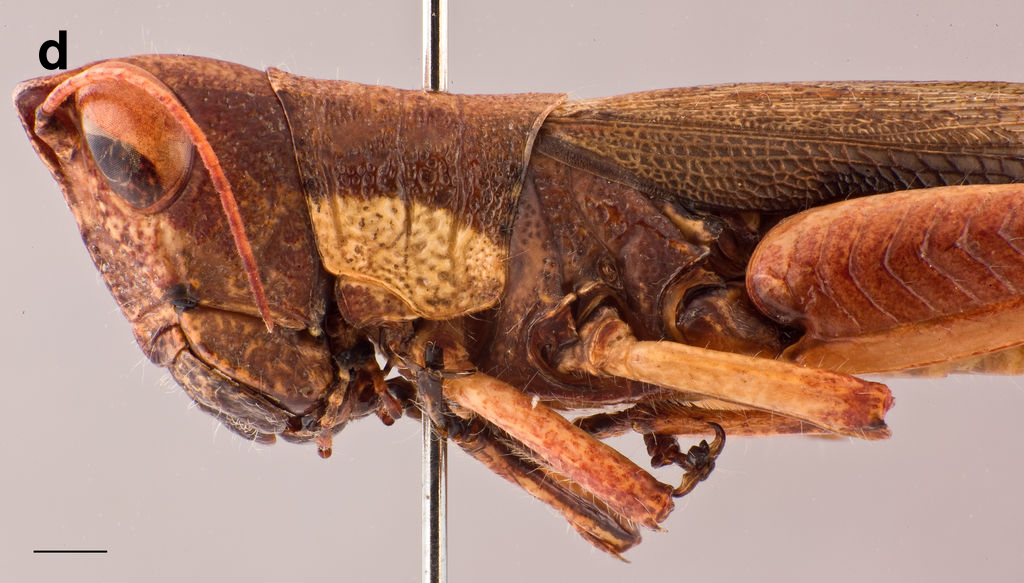
female head and pronotum in lateral view.

**Figure 2e.**
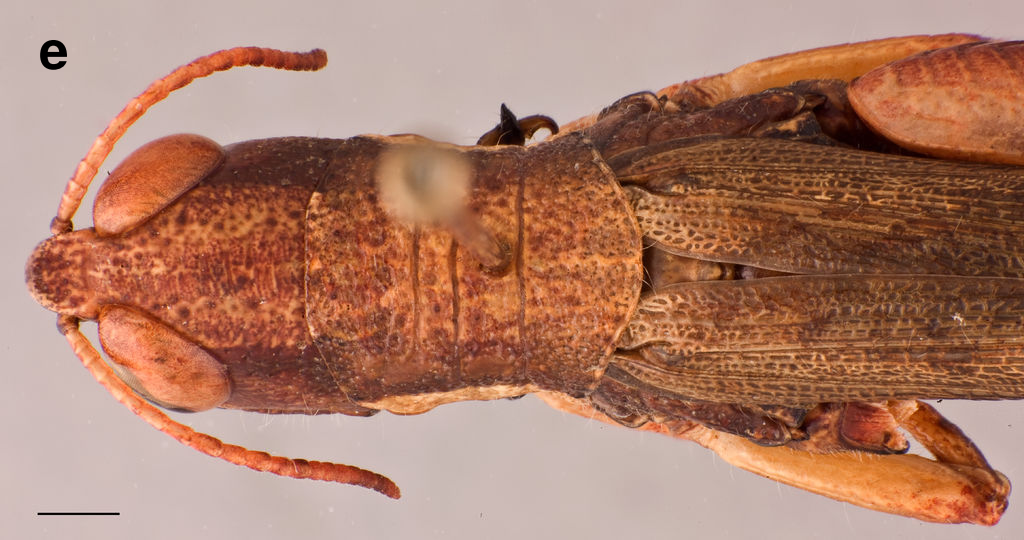
Female head and pronotum in dorsal view.

**Figure 2f.**
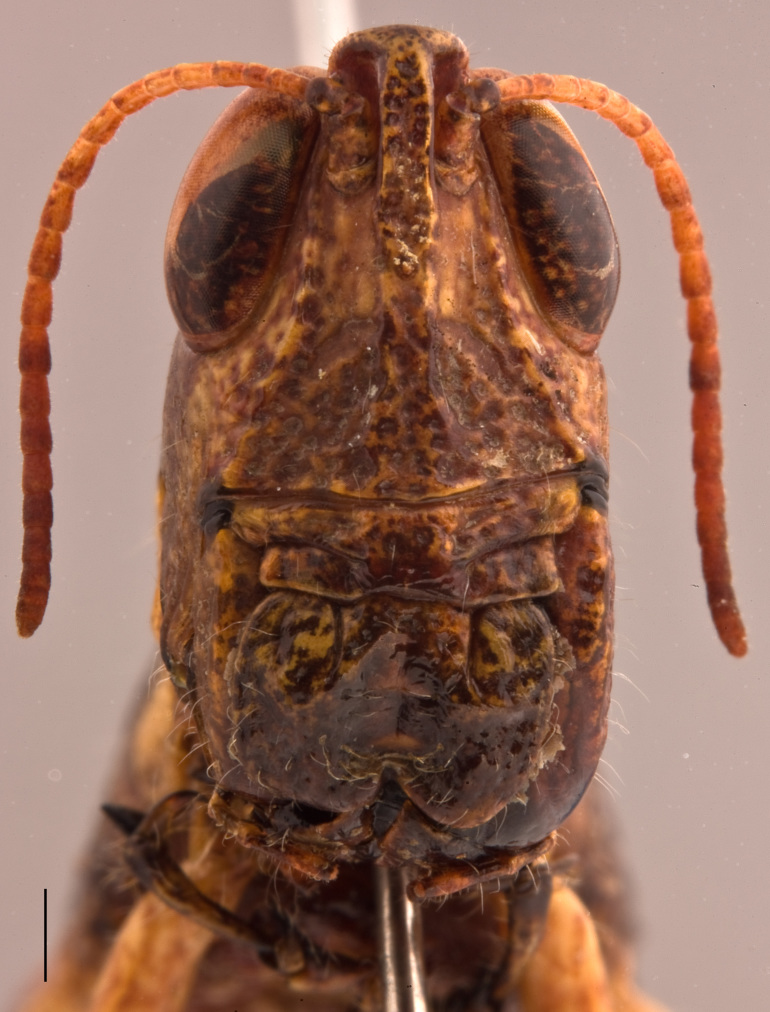
Female face in anterior view.

**Figure 3a.**
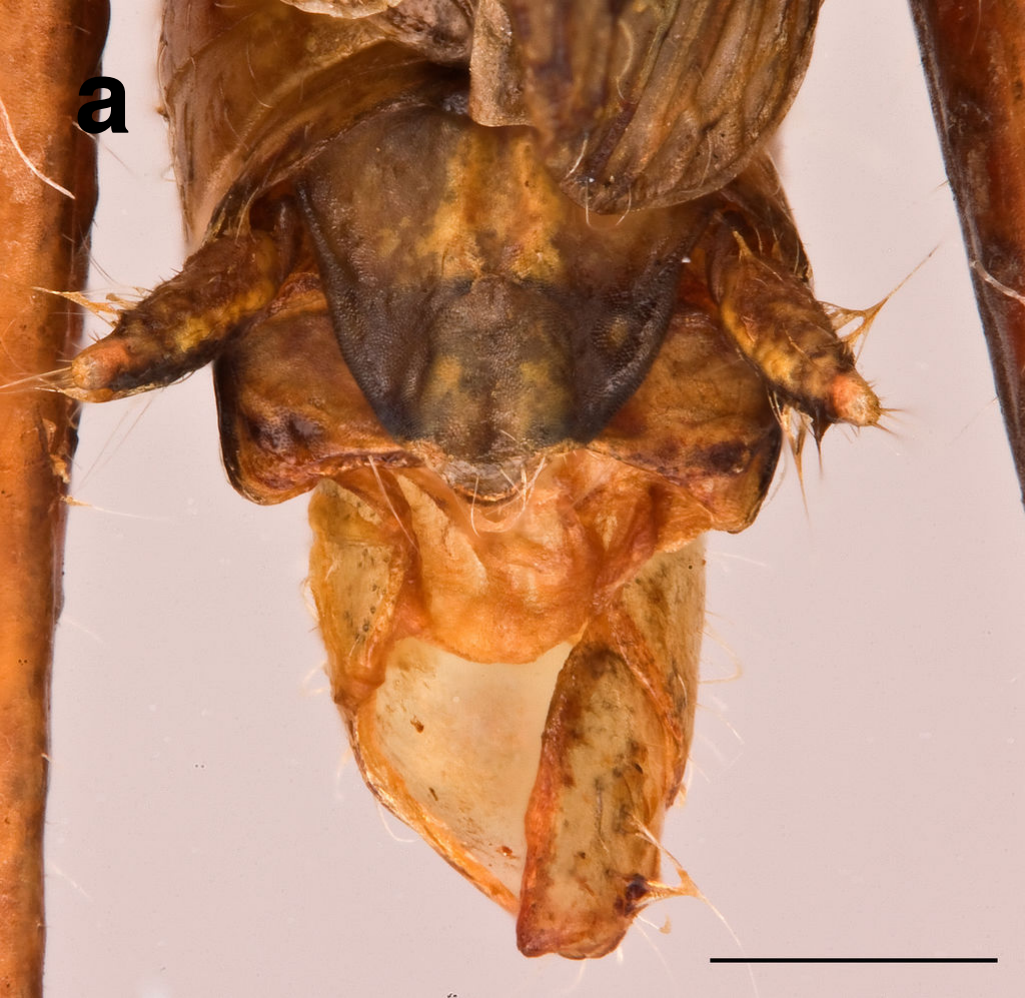
Male abdominal apex in dorsal view.

**Figure 3b.**
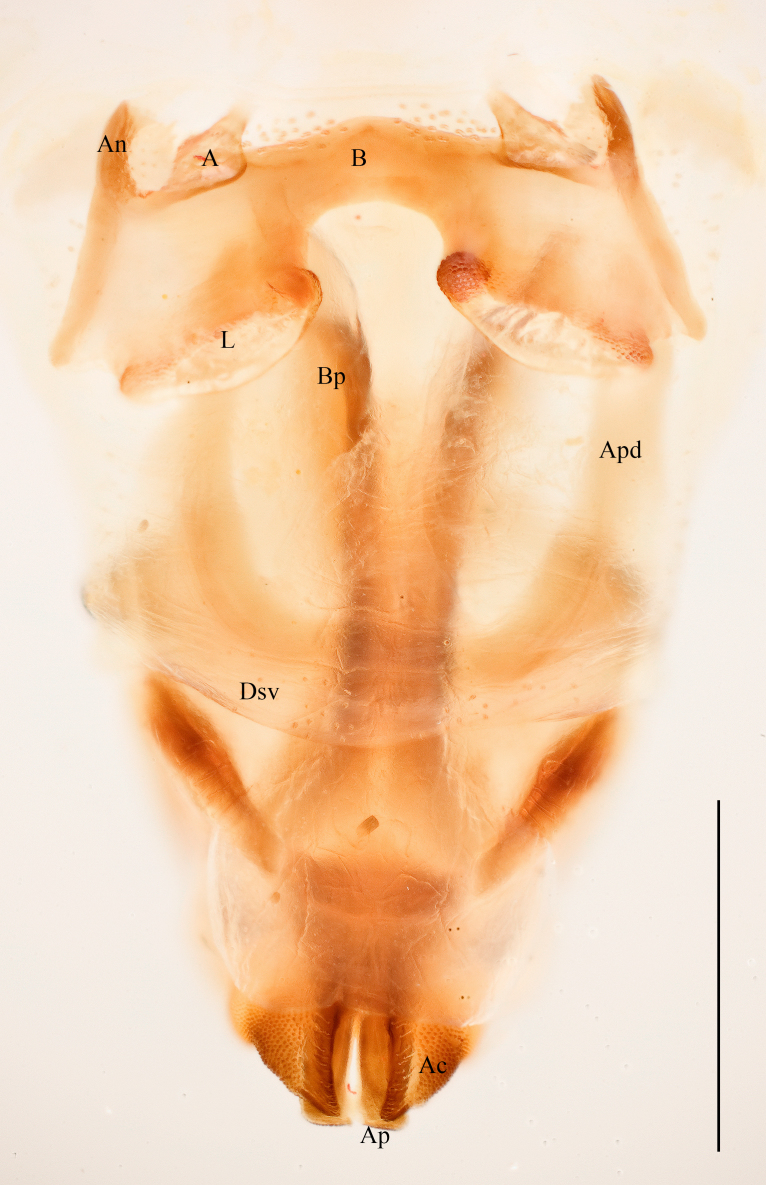
Male phallic complex in dorsal view.

**Figure 3c.**
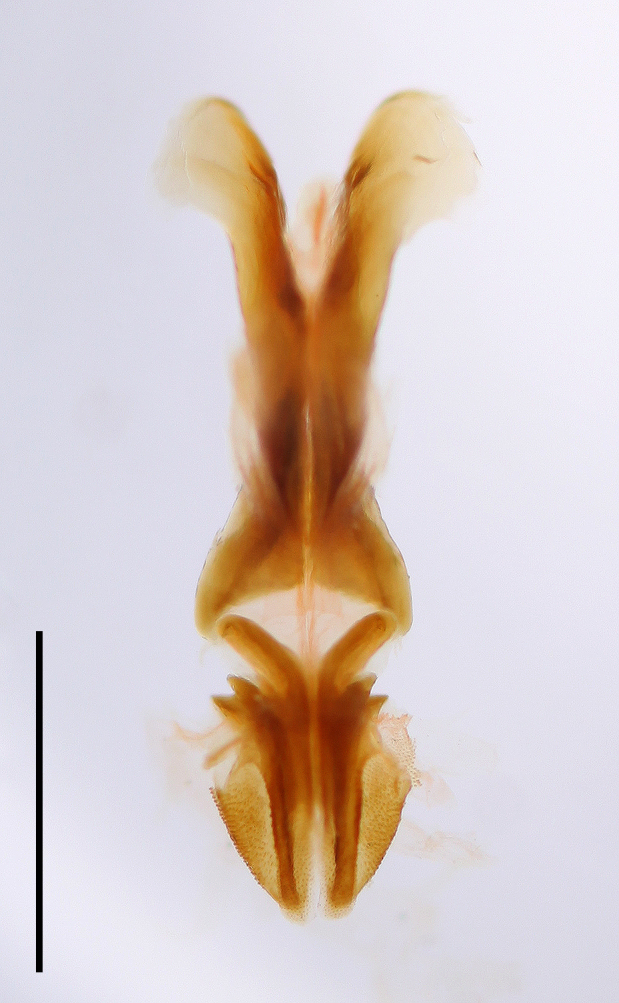
Basal and apical penis valves in dorsal view.

**Figure 3d.**
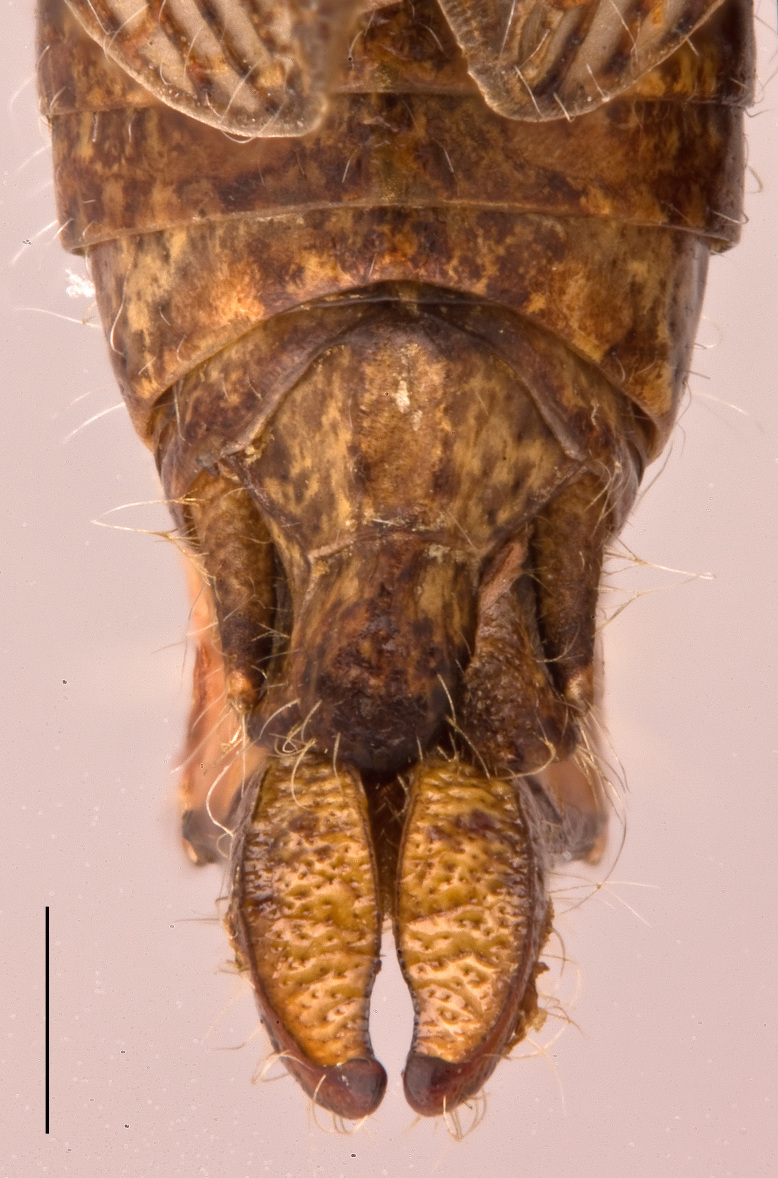
Female abdominal apex in dorsal view.

**Figure 3e.**
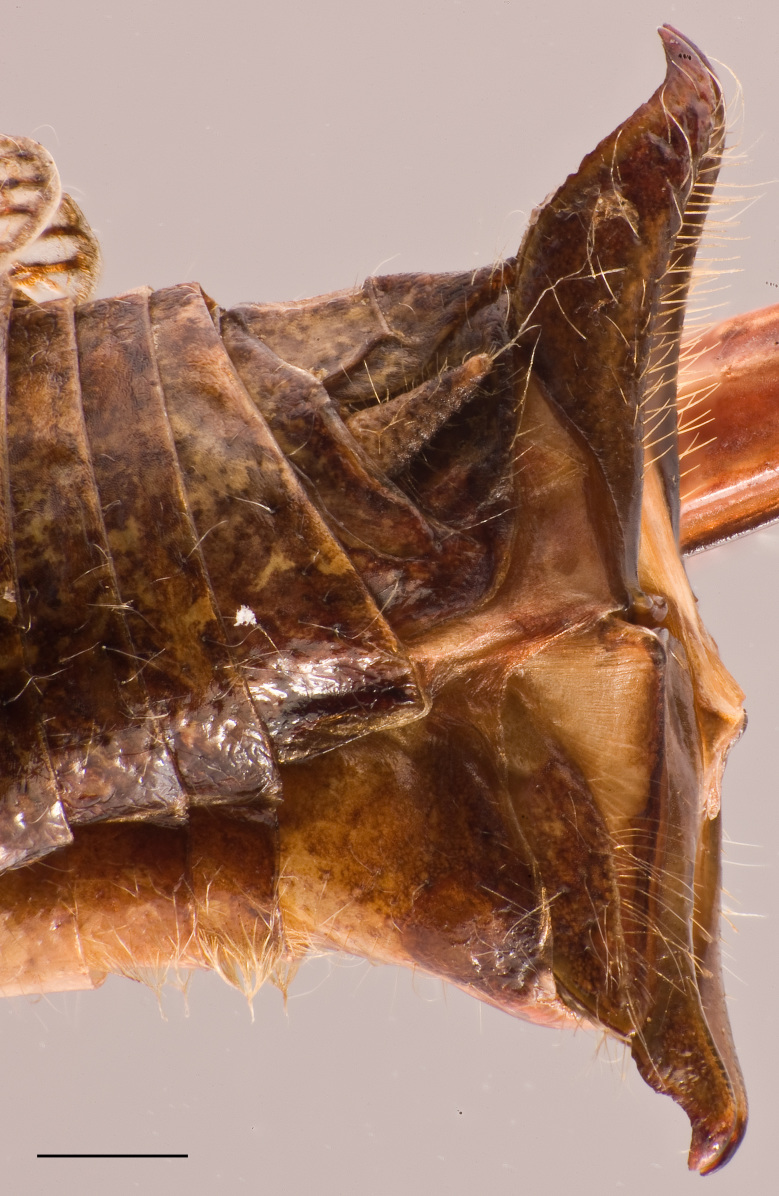
Female abdominal apex in lateral view.

**Figure 3f.**
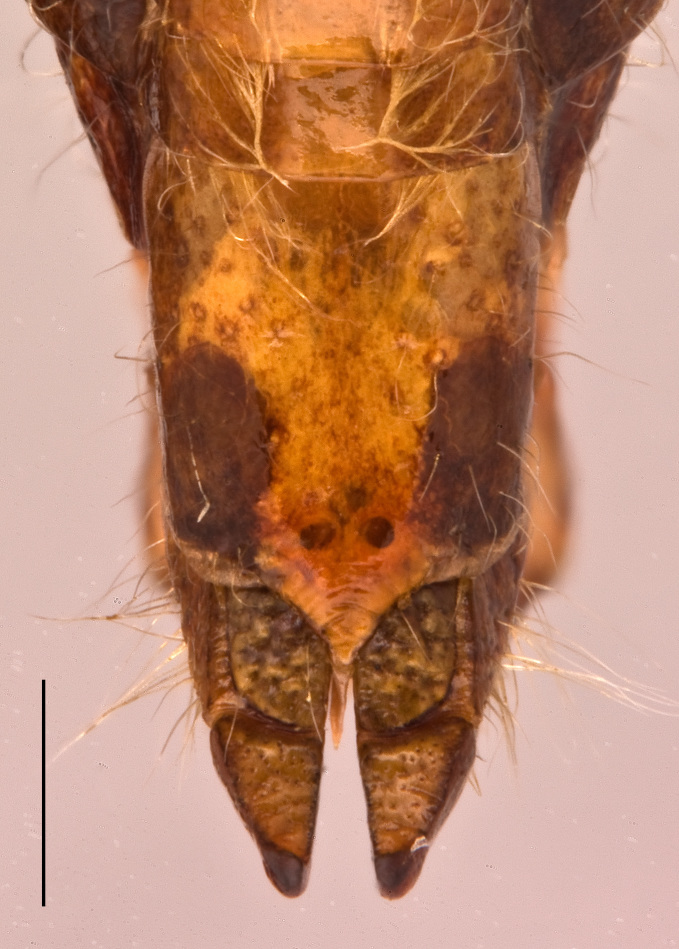
Female abdominal apex in ventral view.
